# Self-perceived care needs and quality of life in people with cognitive impairment during routine care at home: cross-sectional results of the interventional study

**DOI:** 10.1186/s12877-023-03846-w

**Published:** 2023-03-29

**Authors:** Juxia Zhang, Xiaoqin Xu, Xiaoli Zhang, Yuhuan Yin, Jiancheng Wang

**Affiliations:** 1 Clinical Educational Department, Gansu Provincial Hospital, Lanzhou, Gansu 730000 China; 2 Neurology Department, Gansu Provincial Hospital, Lanzhou, Gansu 730000 China; 3 School of Nursing, Gansu University of Chinese Medicine, Lanzhou, Gansu 730000 China; 4 Geriatrics Department, Gansu Provincial Hospital, Lanzhou, Gansu 730000 China

**Keywords:** Quality of life, Physical component summary, Mental component summary, Cognitive impairment, Unmet needs

## Abstract

**Background:**

Cognitive impairment (CI) is one of the most common disabling symptoms in the elderly, and people with CI face a variety of unmet care needs. There is limited evidence on the relationship between unmet needs and quality of life (QoL) of people with CI. The aim of this study is to analyse the current situation of unmet needs and QoL among people with CI, and to explore the correlation between QoL and unmet needs.

**Methods:**

The analyses use baseline data of the intervention trial, which recruited 378 participants to complete the questionnaire including the Camberwell Assessment of Need for the Elderly (CANE), and the Medical Outcomes Study 36-item Short-Form (SF-36). The SF-36 was further gathered into physical component summary (PCS) and mental component summary (MCS). Multiple linear regression analysis was conducted to explore the correlations between unmet care needs and PCS and MCS of SF-36.

**Results:**

The mean score of each of the eight domains of SF-36 was significantly lower than the Chinese population norm. The incidence of unmet needs ranged from 0 to 65.1%. Multiple linear regression results showed that living in rural areas (Beta=-0.16, P < 0.001), having unmet physical needs (Beta=-0.35, *P* < 0.001), and unmet psychological needs (Beta=-0.24, *P* < 0.001) were associated with lower PCS scores, whereas duration of CI > 2 years (Beta=-0.21, *P* < 0.001), unmet environmental needs (Beta=-0.20, *P* < 0.001), and unmet psychological needs (Beta=-0.15, *P* < 0.001) were associated with lower MCS scores.

**Conclusion:**

The main results support the important view that lower QoL scores are associated with unmet needs in people with CI, depending on the domain. Given that the more unmet needs can further worsen QoL, it is recommended that more strategies should be taken, especially for those with unmet care needs, so as to improve their QoL.

## Background

Cognitive impairment is one of the most common and disabling non-motor symptoms in the elderly and also an essential part of the diagnostic criteria for dementia [1]. Progressive cognitive impairment can significantly impact on an individuals’ independence, daily life, and social interactions [1]. The risk of Alzheimer’s disease increases with progression from normal cognition with no amyloid-beta accumulation to early neurodegeneration and subsequently to mild cognitive impairment [2], which is considered a transitional stage between cognitively unimpaired and dementia [3]. It is estimated that about 50 million people worldwide suffer from dementia [3–6]. The prevalence of CI has been reported to be 16.0–22.2% in the United States, 24.1% in South Korea [7], and as high as 38.3% in mainland China [8, 9]. Previous studies have shown the enormous impact that CI can have on the care situation [10]. Lu et al. found that 57% of the people with dementia with severe CI reported that they needed more care than usual [11]. More supported care should be allocated to people with CI. However, these problems are not adequately addressed by healthcare services, resources or support services due to challenges in formal and informal care system [12–14]. Analysis from different countries illustrated that most people with dementia are cared for at home by unpaid, informal caregivers (usually spouses, other family members, or friends) [4], with one-third of them are estimated to live alone [14]. A study assessing the needs of people with middle-stage dementia from eight European countries showed higher levels of psychological distress, need for daytime activity, company and information [15]. Moreover, another study reported that 58% of people with CI had unmet needs in advance care planning [16]. A study in German reported that more than 90% of people with dementia had three or more unmet needs, most of which occurred in the area of “nursing treatment and care” [17]. A previous study by our group showed that people with CI mainly have unmet needs in caring for someone, looking after the home and self-care [18], which means these people still face a long list of care needs [13, 19]. Since there are no well-established formal and informal care pathways for people with CI, even in developed settings, addressing their care needs and designing services centres around their needs, to improve their QoL, becomes an urgent public health priority [20].

Quality of life is an increasingly common outcome of the successful management of disease [21], it is also an important indicator in research on dementia, particularly in determining the burden of care needs due to disease and disabilities [22, 23]. Currently, there are no curative therapies for dementia [24], therefore the primary goal of CI management is to maximize QoL by helping people cope with their functional and cognitive performance, delaying the decline in activities of daily living, and assisting patients and caregivers to obtain the necessary care and services [25].

Until 2019, people over 65 years old in China account for 18% of the global population [5], by 2060, there will be more than 300 million [6]. To support aging, the Chinese government encourages a ‘90/7/3’ pattern of the eldercare system, with 90% be cared for at home, 7% in communities, and 3% in institutions [26]. Although the government has made great efforts in establishing a multi-dimensional geriatric care system, it is estimated that among the over 40 million people with different degrees of care needs, more than 90% are not yet cared for [4, 8]. Therefore, in order to address those issues effectively, it is necessary to identify and understand the QoL from the perspective of people with CI, while understanding their care needs in living and interactions. To date, there is limited evidence on the association between the care needs and QoL of people with CI in China. Therefore, the aim of this study is to bridge this gap by addressing the following questions: (1) What is the QoL of people with CI living at home in China, and (2) How does unmet care needs relate to their QoL?

## Methods

### Study design

This analysis is part of the ongoing study (The Construction, Construction and Empirical Study of home-based Supportive Care Program for patients with cognitive impairment and their caregivers) which compare the effectiveness of an intervention in a group receiving the intervention (intersectoral care management) with a control group receiving “usual care”. The design of the study has been published in a Chinese journal and the data of care needs assessment has been published in more detail elsewhere [18]. The present analysis is based on cross-sectional data of all participants at baseline.

### Participants

Participants were recruited from the memory clinics at Gansu Provincial Hospital between January to April 2019, the clinics sees 5–10 new and 20–30 follow up patients each week. The clinic team consists of a neurologist, nurses, medical students, and psychologists offering services of full screening, diagnosis, and medical treatment. For All clinic patients have their cognitive level screened using a brief neuropsychological test, the Mini Mental State Examination (MMSE) [27]. The range of MMSE scores is 0 to 30 points. Higher MMSE scores indicate better cognitive function [28, 29]. Since MMSE scores are easily influenced by educational level, the following cut off scores for defining CI for different levels of education were used: ≤17 points for illiterate people (< 1 year of education), ≤ 20 points for people with primary school (1–6 years of education), and ≤ 24 points for people with middle school or higher (> 6 years of education) [29, 30]. For enrollment, one specially trained staff identified possible participants who met following inclusion criteria: 1) ≥ 65-year old; 2) MMSE ≤ 17 points for illiterate people, MMSE ≤ 20 points for people with primary school, and MMSE ≤ 24 points for people with middle school or higher; 3) Living at home for at least 1 year; 4) Informed and consenting to participate in this study. In order to ensure the reliability of self-reported data, the exclusion criteria are as follows: (1) people with hearing impairment, visual impairment and communication difficulties; (2) serious psychiatric problems; (3) behavior disorders (severe aggression, behavior disturbing group work, lack of control or impulsive behavior); (4) People with other terminal diseases that have a shorter expected survival period. Since the treatment and care of older people with CI is often dependent on informal caregivers, participants were asked to name their informal caregivers (e.g., spouse, child, friend). A total of 550 patients were screened. Of them, 406 patients were eligible and finally 378 individuals gave consent to participate in the study.

### Instrument

#### Socio-demographic and clinical characteristics

Socio-demographic characteristics: age, gender (male and female), education level (illiteracy, primary school and middle school or higher), living status (with spouse, with others), residence (rural or urban). The duration of CI was defined as the interval between the time of first screening for CI and the time that the study started. As the overall diagnosis time was not normally distributed, we divided the diagnosis time into two groups (≤ 2 years and > 2 years) based on the approximate value of the median diagnosis time (2.08 years) for better statistical analysis. Similarly, we divided the MMSE scores into two groups (≤ 21 scores and > 21 scores) based on the median MMSE scores (21 scores).

#### Measurement of care needs

Care needs were identified using the Camberwell Assessment of Need for the Elderly (CANE) which is designed to map the needs and amount of help (received and needed) for older people [18]. It is a structured, multi-dimensional needs assessment scale that covers the environmental, physical, and psychosocial domains [31]. In the study, a met need was defined as receiving sufficient help to solve or reduce the problem, whereas an unmet need was defined as a lack of help or insufficient help. In this study, responses are rated on a scale where no need was scored 0, met need was scored 1 and unmet need was scored 2. The total possible score ranged from 0 to 48, with a higher score indicating a greater amount of unmet needs. Meanwhile, based on the results for each individual, the numbers of met and unmet needs were calculated, as well as the number of all needs as a sum of met and unmet needs. Cronbach’s alpha of the CANE was 0.946 [18], which indicated that the reliability of the CANE was good. CANE has been shown to be suitable for elderly people with different levels of cognitive function [18, 32].

#### Measurement of QOL

QOL was measured using the widely used Chinese version of the Medical Outcomes Study 36-Item Short Form (SF-36) [33], which includes eight domains: Physical Functioning (PF), Role Physical (RP), Bodily Pain (BP), General Health (GH), Vitality (VT), Social Functioning (SF), Role Emotional (RE), and Mental Health (MH). Based on the response to each item comprising the eight domains and using a z-score transformation, the scores of each domain were calculated [34]. Item responses from each domain were summed and converted into a standard range of 0 to 100, as shown in the following formula, the higher scores define better QOL.

$$Score = \frac{{{\text{actual score - the lowest possible score of the subscale}}}}{{\text{the highest score of the subscale - the lowest score of the subscale}}} \times 100\%$$

Scoring norms for the Chinese version of the SF-36 were given which have been proven to be valid [8]. Then these eight domain scores were compared to Chinese normative data [35]. The eight domains were further clustered into two total scores: Physical component summary (PCS) (comprised of PF, RP, BP, and GH domains) and Mental component summary (MCS) (comprised of the VT, SF, RE, and MH domains) [23].

### Ethics approval

This study was approved by the Ethics Committee of Gansu Provincial Hospital (2020 − 234). All subjects were informed and signed informed consent. All clinical investigations were conducted according to the principles expressed in the Declaration of Helsinki.

### Procedure

A researcher first screened the follow-up list and identified eligible potential participants in the memory clinic. The study assistants communicated with the participants in a language the older adults understood and in a manner acceptable to them, and obtained consent from the participants when they had the cognitive ability to consent. If the participant does not have the cognitive ability to consent to participate in the study, the caregiver’s consent must be sought. The researchers guaranteed that they could quit at any stage without giving reason. For those who met the criteria and agreed to participate, the researchers asked them to sign an informed consent form. The principal investigator then conducted face-to-face interviews using structured questionnaires and recorded participants’ responses. Other researchers reviewed the participants’ medical records with their consent to obtain their clinical characteristics and duration of CI.

### Statistical analysis

Statistical analyses were undertaken using the SPSS 21.0 software. First, variables were summarized using descriptive statistics, categorical variables were represented by absolute (n) and relative (%) frequencies. Normally distributed numerical variables were represented by mean ($\stackrel{-}{\text{x}}$) and standard deviation (SD), numerical variables with skewed distribution (duration of CI, MMSE scores) were represented by median (M) and interquartile range (IQR = Quartile 3-Quartile 1). For the analyses, we used the total number of needs (both met and unmet) and needs scores as an indication of requiring care where higher scores indicate higher care needs. The mean score for each domain of the SF-36 was calculated and compared with the norms from the general Chinese population using a t-test. Pearson correlation analysis, one-way ANOVA, and t-test were performed to preliminarily explore the correlation between general characteristics, unmet needs, and PCS and MCS. Then, multiple linear regression analyses were performed to determine the potential predictors (general characteristics and care needs) of PCS and MCS. Dependent variables were PCS and MCS scores (numerical variables with normal distribution). The independent variable is the variable with *P* < 0.1 in the univariate analysis. No significant violation of normality was found in assessments of the residuals. During multivariable modeling, the variance inflation factor (VIF) were used to detect multicollinearity. Any predictor with a VIF above 10 was excluded from the final model. For all analyses, the statistical significance level was set at *P* < 0.05.

## Results

### General characteristics of participants

As shown in Table 1, the mean age was 69.40 ± 11.59. Of participants, 57.14% were males, and 39.68% were living with spouses. Nearly 70% of subjects were urban residents, and about half of the participants had a duration of CI for more than 2 years. Overall, the median MMSE score for all participants was 21 (IQR: 17–23).

**Table 1:** Association between QoL, general characteristics and unmet needs (N = 378)

Characteristics		N (%)/$\stackrel{-}{x}$±SD/M(IQR)	PCS		MCS	
			$\stackrel{-}{x}$±SD	*P*	$\stackrel{-}{x}$±SD	*P*
Age (years)		69.40 ± 11.59		< 0.001		0.326
Gender				0.059		0.128
	Male	216 (57.14)	53.53 ± 14.26		54.93 ± 15.07	
	Female	162 (42.86)	56.39 ± 14.80		57.55 ± 17.57	
Education level				0.286		0.041
	Illiteracy	57 (15.08)	52.17 ± 15.67		54.78 ± 18.22	
	Primary school	34 (8.99)	53.75 ± 11.19		49.87 ± 13.82	
	Middle school or higher	287 (75.93)	55.39 ± 14.65		57.04 ± 15.94	
Living status				0.253		0.392
	With spouse	150 (39.68)	55.84 ± 15.65		55.17 ± 16.04	
	With others	228 (60.32)	54.04 ± 13.76		56.63 ± 16.35	
Residence				< 0.001		0.125
	Rural	117 (30.95)	48.81 ± 16.03		54.76 ± 18.78	
	Urban	261 (69.05)	57.42 ± 12.99		57.34 ± 16.35	
Duration of CI (years)		2.08 (1.00-3.17)		0.445		< 0.001
	≤ 2	175 (46.30)	55.37 ± 14.45		61.09 ± 17.19	
	> 2	203 (53.70)	54.23 ± 14.65		51.71 ± 13.99	
MMSE scores		21 (17.00–23.00)		0.005		< 0.001
	≤ 21	190 (50.26)	52.68 ± 15.48		52.50 ± 16.63	
	> 21	188 (49.74)	56.85 ± 13.25		59.64 ± 15.01	
Participants’ needs						
	Environmental needs	5.71 ± 2.14		< 0.001		< 0.001
	Physical needs	5.17 ± 2.14		< 0.001		< 0.001
	Psychological needs	3.44 ± 1.86		< 0.001		< 0.001
	Social needs	3.27 ± 1.02		0.02		0.001
PCS		54.76 ± 14.58				
MCS		56.05 ± 16.27				

M (IQR): median (Interquartile range = Quartile 3-Quartile 1); MMSE: Mini Mental State Examination;

PCS: Physical component summary; MCS: Mental component summary.

### Self-reported unmet care needs

Most unmet needs were environmental (5.71 ± 2.14), followed by physical (5.17 ± 2.14), psychological (3.44 ± 1.86), and social needs (3.27 ± 2.02) (shown in Table 1). The incidence of unmet needs ranged from 0 to 65.1%, with the incidence of unmet needs in the three areas of caring for others, caring for family, and self-care exceeding 50% (shown in Table 2).

**Table 2:** Descriptions of reported met and unmet needs for CANE items (N = 378)

Items	Area	No need n (%)	Full/Partiall met needs, n(%)	Unmet needs, n (%)
Environmental needs	Accommodation	0 (0.00)	357 (94.44)	21 (5.56)
	Looking after the home	96 (25.40)	42 (11.11)	240 (63.49)
	Food	318 (84.13)	21 (5.56)	39 (10.32)
	Money/budgeting	147 (38.89)	204 (53.97)	27 (7.14)
	Benefits	90 (23.81)	234 (61.90)	54 (14.29)
	Caring for someone else	93 (24.60)	39 (10.32)	246 (65.08)
Physical needs	Physical health	6 (1.59)	306 (80.95)	66 (17.46)
	Medication	18 (4.76)	294 (77.78)	66 (17.46)
	Eyesight/hearing/communication	240 (63.49)	81 (21.43)	57 (15.08)
	Mobility/falls	150 (39.68)	120 (31.75)	108 (28.57)
	Self-care	102 (26.98)	54 (14.29)	222 (58.73)
	Continence	312 (82.54)	48 (12.70)	18 (4.76)
Psychological needs	Psychological distress	129 (34.13)	210 (55.56)	39 (10.32)
	Memory	90 (23.81)	225 (59.52)	63 (16.67)
	Behavior	138 (36.51)	156 (41.27)	84 (22.22)
	Alcohol	372 (98.41)	6 (1.59)	0 (0.00)
	Deliberate self-harm	375 (99.21)	3 (0.79)	0 (0.00)
	Inadvertent self-harm	360 (95.24)	15 (3.97)	3 (0.79)
	Psychotic symptoms	195 (51.59)	132 (34.92)	51 (13.49)
Social needs	Company	138 (36.51)	120 (31.75)	120 (31.75)
	Intimate relationships	63 (16.67)	147 (38.89)	168 (44.44)
	Daytime activities	105 (27.78)	171 (45.24)	102 (26.98)
	Information	243 (64.29)	102 (26.98)	33 (8.73)
	Abuse/neglect	324 (85.71)	33 (8.73)	21 (5.56)
Mean needs		18.52 ± 5.40		

### QoL of participants

**Fig. 1:**
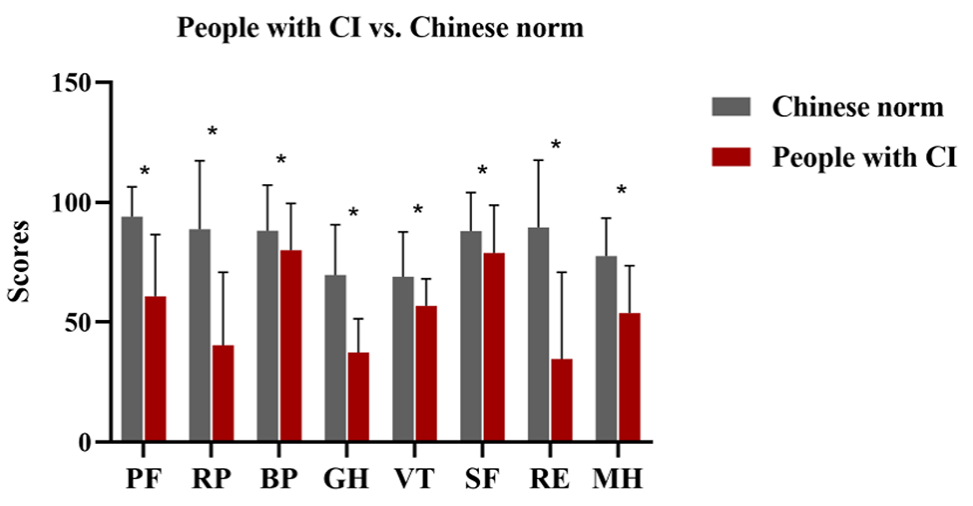
Mean scores of SF-36 for people with CI vs. Chinese norm. PF, Physical Functioning; RP, Role Physical; BP, Bodily Pain; GH, General Health; VT, Vitality; SF, Social Functioning; RE, Role Emotional; MH, Mental Health. *P < 0.05

The mean score for eight domains of SF-36 was shown in Fig. 1. The mean score of each of eight domains (PF, RP, BP, GH, VT, SF, RE, and MH) was significantly lower than the Chinese norm, the biggest differences were in RP and RE, which were less than 50% of the norm. The mean scores for the PCS and MCS were 54.76 ± 14.58 and 56.05 ± 16.27, respectively.

### Correlations between QoL and unmet needs

Multiple linear regression results showed that living in rural areas (Beta=-0.16, *P* < 0.001), unmet physical needs (Beta=-0.35, *P* < 0.001), and unmet psychological needs (Beta=-0.24, *P* < 0.001) were associated with lower PCS scores (shown in Table 3).

Duration of CI > 2 years (Beta=-0.21, *P* < 0.001), unmet environmental needs (Beta=-0.20, *P* < 0.001), and unmet psychological needs (Beta=-0.15, *P* < 0.001) were associated with lower MCS scores (shown in Table 4).

**Table 3:** Multiple linear regression analysis between general characteristics, needs scores and PCS.

Factors		B (95%CI)	Beta	t	*P*	
Age		−0.04 (−0.16, 0.08)	−0.03	−0.691	0.490	
Gender						
	Male	Ref				
	Female	2.33 (−0.04, 4.70)	0.08	1.933	0.054	
Residence						
	Urban	Ref				
	Rural	−5.13 (−7.79,−2.47)	−0.16	−3.797	< 0.001**	
MMSE scores						
	≤ 21	Ref				
	> 21	−1.69 (−4.16, 0.78)	−0.06	1.343	0.180	
Environmental needs		−0.63 (−1.33, 0.07)	−0.09	−1.786	0.078	
Physical needs		−2.36 (−3.15,−1.57)	−0.35	−5.877	< 0.001**	
Psychological needs		−1.88 (−2.68,−1.08)	−0.24	−4.613	< 0.001**	
Social needs		0.17 (−0.43, 0.77)	−0.02	0.546	0.585	

B: Unstandardized Coefficients; 95% CI, 95% confidence interval for B; The value Beta indicates Standardized Coefficients; Ref: Reference group; **P < 0.01;

**Table 4:** Multiple linear regression analysis between general characteristics, needs scores and MCS

Factors		B (95%CI)	Beta	t	*P*
Education					
	Illiteracy	Ref			
	Primary school	-0.72 (-2.30, 0.85)	−0.05	-0.903	0.367
	Middle school or higher	-0.36 (-1.23, 0.52)	−0.05	-0.796	0.427
Duration of CI (years)					
	≤ 2	Ref			
	> 2	-6.81 (-9.90, -3.72)	-0.21	-4.331	< 0.001**
MMSE scores					
	≤ 21	Ref			
	> 21	3.10 (-0.24, 6.44)	0.10	1.828	0.068
Environmental needs		-1.46 (-2.37, -0.54)	-0.20	-3.141	0.002**
Physical needs		-0.62 (-1.55, 0.32)	-0.08	-1.292	0.197
Psychological needs		-1.28 (-2.28, -0.29)	-0.15	-2.541	0.011*
Social needs		-0.34 (-1.10, 0.43)	-0.04	-0.867	0.387

B: Unstandardized Coefficients; 95% CI, 95% confidence interval for B; The value Beta indicates Standardized Coefficients; MMSE: Mini-Mental State Examination; Ref: Reference group; *P < 0.05, **P < 0.01.

## Discussion

The present study provided important information on specific predictors of PCS and MCS of SF-36 in people with CI, with a particular focus on the contribution of their unmet needs. Our study showed that compared with the general Chinese population, people with CI reported worse QoL scores for all domains, particularly for role-physical and role-emotional problems, which were less than 50% of the general Chinese population, similar results have been found in the Netherlands and the UK [33, 36]. The main results support the important view that lower QoL scores for people with CI are associated with unmet needs. In this study, the incidence of unmet needs ranged from 0 to 65.1%, with more than 50% having unmet needs in caring for others, caring for family, and self-care.

The unmet environmental needs were significantly related to MCS, which were mainly reflected in taking care of the family and caring for others, and more than 60% of the participants’ needs were not met in these two aspects. Some symptoms of CI may prevent the elderly from participating in home care activities (such as cleaning rooms, cooking, etc.), thus diminishing their role in caring for the family [18]. This phenomenon may indicate that people with CI in China are more concerned about the role of taking care of the family rather than their own psychological and social functions. In addition, this study showed that duration of CI was negatively correlated with MCS. In our study, more than half of the participants had CI for more than 2 years. The likely explanation is that in the early stages of CI, people are able to maintain most of their daily life and activities. The longer the duration of CI, the greater the participants’ symptom progression and subjective burden [37], the more assistance they need to perform many activities of daily living, and in severe CI most people become almost or completely dependent on their caregivers [38, 39]. Unmet care needs can have a negative impact on mood leading to a decline in psychological quality of life. Therefore, it is not surprising to observe the negative impact of a linger history of CI on quality of life.

Although there are relatively few unmet psychological needs compared to other fields, surprisingly, multiple linear regression results showed that psychological needs are a common predictor of PCS and MCS. This may be mainly manifested in behavioral, memory, and psychiatric symptoms. Although the incidence of unmet needs in these areas was not high, memory impairment is the most common symptom of people with CI [40, 41]. Dickson et al. found that memory and attention deficits can lead to forgetfulness and poor learning, which can impair patients’ adherence to treatment [42]. Memory impairment may also inhibit the learning and retention of information needed to maintain the stability of the disease, such as remembering the dosage and time of medication [43]. Furthermore, people with memory impairment may not recognize symptoms of acute compensatory dysregulation, which may impede early hospitalization [43]. In addition, people with CI may exhibit behavioral disorders and personality changes [44]. When patients are unable to verbally express unmet expectations, they may express these feelings in an agitated manner [45, 46], which reflects both a reduction in service provision and the fact that most care assistance is dependent on family caregivers [47], resulting in more unmet needs and affecting the QoL of people with CI.

Our study also showed that unmet physical needs are significantly related to PCS, mainly reflected in the unmet need for self-care. Nearly 60% of participants in our study had unmet self-care needs. Vellone et al. found that impairments in memory and simple attention may affect confidence in self-care in people with CI [48]. Unlike nursing homes or other day care facilities, people with cognitive disabilities who live at home receive most of the help they receive from informal caregivers (family and friends) [49, 50]. In this study, 60% of participants lived with someone other that a spouse. Most young people may not be able to meet some of the needs of people with CI due to busy work or heavy family burdens [18]. This means that people with CI often need to care for themselves out of necessity, but may have impaired ability to self-care because of CI or other co-morbidities associated with aging, thus reducing their QoL further. In addition, we found that people living in rural areas had lower PCS scores. As demographic trends change, traditional values erode and geographical mobility increases, fewer young family members in rural areas live nearby to support the elderly [34]. As a result, people with CI have more unmet needs, which affects their QoL. However, in our study, the lack of a statistically significant association between unmet social needs and QoL may have related to a wide range of CI amongst participants.

Studies have shown that physical activity or exercise programs could help maintain mobility, improve mood, and reduce behavioral disorders [51–53]. Thus, caregivers can organize more group activities, through conversation and social activities to reduce the social isolation of people with CI. At the same time, caregivers should also respect their dignity, allow patients to express emotions, provide emotional support, empower patients to meet their psychological needs, so as to improve the QOL of people with CI. In addition, it is recommended that more people providing care for people be CI be trained. There is a need to create opportunities for caregivers to participate in workshops with health professionals. Meanwhile, it is necessary to establish day-care centers in the community with the participation of multidisciplinary teams to provide people with CI with social, psychological, and other complementary measures to meet the basic physical needs of people, so as to improve the quality of life of patients with CI.

## Limitations

There are some limitations for the study. First, the data were collected from a single clinic, the findings may have limited generalizability, furthermore, Multiple-centered samples are needed to support our findings. Second, our study only evaluated the needs and QoL from the perspective of people with CI, and did not include the needs of caregivers and staff, further comprehensive analysis is needed in the future. Third, in this study, participants’ cognitive function was assessed only by MMSE. The included participants had a wide range of cognitive levels and were not further assessed for progression to dementia. We will study this issue further in the future.

## Conclusion

This study provides reference for clinical decision makers to formulate interventions for people with CI. The main results support the important view that lower QoL scores for people with CI are associated with unmet needs, depending on the domain. Given that the unmet needs of people with CI can worsen QoL, it is recommended that training for people caring for people with CI be offered, so as to improve and support the care they provide. Meanwhile, it is necessary to establish day-care centers in the community with the participation of multidisciplinary teams to provide CI person with social, psychological, and other complementary measures to meet the basic physical needs of patients, so as to improve the quality of life of people with CI.

## Data Availability

The datasets used and/or analysed during the current study available from the corresponding author on reasonable request.

## Abbreviations

AD: Alzheimer’s disease
CI: Cognitive impairment
CANE: Camberwell Assessment of Need for the Elderly
MMSE: Mini-Mental State Examination
QoL: Quality of Life
SF-36: Medical Outcomes Study 36-Item Short Form
PCS: Physical component summary
MCS: Mental component summary
PF: Physical Functioning
RP: Role Physical
BP: Bodily Pain
GH: General Health
VT: Vitality
SF: Social Functioning
RE: Role Emotional
MH: Mental Health.
